# Enhancing Short Track Speed Skating Performance through Improved DDQN Tactical Decision Model

**DOI:** 10.3390/s23249904

**Published:** 2023-12-18

**Authors:** Yuanbo Yang, Feimo Li, Hongxing Chang

**Affiliations:** 1 School of Information Science and Technology, Beijing Forestry University Beijing, Beijing 100107, China; yangyuanbo2021@bjfu.edu.cn; 2 Institute of Automation Chinese Academy of Sciences Beijing, Beijing 100190, China; hongxing.chang@ia.ac.cn

**Keywords:** short track speed skating, deep reinforcement learning, decision-making method, deep Q-network, competition performance improvement

## Abstract

This paper studies the tactical decision-making model of short track speed skating based on deep reinforcement learning, so as to improve the competitive performance of corresponding short track speed skaters. Short track speed skating, a traditional discipline in the Winter Olympics since its establishment in 1988, has consistently garnered attention. As artificial intelligence continues to advance, the utilization of deep learning methods to enhance athletes’ tactical decision-making capabilities has become increasingly prevalent. Traditional tactical decision techniques often rely on the experience and knowledge of coaches and video analysis methods that require a lot of time and effort. Consequently, this study proposes a scientific simulation environment for short track speed skating, that accurately simulates the physical attributes of the venue, the physiological fitness of the athletes, and the rules of the competition. The Double Deep Q-Network (DDQN) model is enhanced and utilized, with improvements to the reward function and the distinct description of four tactics. This enables agents to learn optimal tactical decisions in various competitive states with a simulation environment. Experimental results demonstrate that this approach effectively enhances the competition performance and physiological fitness allocation of short track speed skaters.

## 1. Introduction

Short track speed skating has been a traditional competition of the Winter Olympics since its inception in 1988. With the continuous improvement of intelligence, it is more important to use advanced technology to improve the competitive level of short track speed skaters. The traditional way to improve the performance of short track speed skaters is to analyze and guide athletes at the level of physiology and mechanics of human motion. Felser et al. [1] study maximum voluntary contraction (MVC) strength of the leg muscles and time of 17 young short track speed skaters and finally concludes that stabilizing ankle joint and knee is the main factor to ensure the strength quality. Deguire et al. [2] propose that hypoxic repetitive sprint training can improve repetitive sprint ability and high-intensity performance for national short track speed skaters. Noorbergen et al. [3] study the data of short track speed skaters at 500 m and 1000 m in different seasons in order to provide tactical ideas at the start of the race and each lap. Hext et al. [4] provide a new analysis perspective for short track speed skating by serialization analysis tactical positioning. Knobbe et al. [5] model 15 years of records of elite speed skating team LottoNL-Jumbo in order to obtain potential sources of knowledge and thereby improve training earnings. The above articles prove that the integration of data analysis methods into short track speed skating can effectively improve the performance of athletes.

Traditional tactical decision-making methods are often completed by manual guidance of coaches on training and competition data [6,7,8,9]. Through repeated analysis of the competition video and data of the athletes, the coaches make tactical plans for the athletes based on their own experience, which increases the workload of the coaches. There are few related works that combine deep learning with tactical decision-making in short-track speed skating. Changpeng et al. [10], respectively, analyze and compares the decision-making ability of short track speed skating agent based on the BP neural network and decision tree. Although the result shows that the agent has improved the ability to complete the race, the output of the model is excessively dependent on the manually operated agent trajectory data and the fuzzy interpretation of the curve section causes the agent to repeat the same actions subject to other constraints. Yang et al. [11] use Double Deep Q-network (DDQN) [12] to learn the short track speed skating trajectory of the agent in the simulation environment to improve its tactical decision-making ability. This method guides the agent to learn the tactics with the highest cumulative reward by setting a reward function, so as to make tactical decision planning for the agent in different competition states. However, the simulation environment designed in that paper lacks many physical restrictions, and the complex reward function leads to excessive computation and slow convergence time in the learning process.

Therefore, in order to solve the above problems and further improve the decision-making efficiency of agents, we propose a short track speed skating tactical decision-making model based on deep reinforcement learning. Firstly, we build a simulation environment for the speed skating competition venue, so as to accurately simulate the physical properties of the skating venue, the multiple types of physiological data for athletes and the rules of the short track speed skating competition. Secondly, in order to fully leverage the strengths of reinforcement learning in addressing decision-making problems, we propose the improved DDQN tactical decision model. The strategies explored based on this approach could allow the agent to take the appropriate tactical actions at each step to maximize the cumulative reward and to achieve the effect of improving the performance of the athletes [13]. The main contributions of this work can be summarized as follows:

- We propose a tactical decision model based on DDQN and improve a more scientifically rigorous reward function. Therefore, agents can more efficiently explore and learn tactical decision-making behaviors in different competitive states in the simulation environment.
- We model a real short track speed skating competition, which effectively simulates the rules of a 500-meter short track speed skating event, the physiological data of the athletes, and the information about the competition venue.
- We use the competition data from 16 groups of real athletes for training and testing. Our approach can effectively improve tactical decision-making capabilities. Experimental results demonstrate the effectiveness of our proposed method.

## 2. Related Work

With the popularity of recommendation algorithms, the use of deep reinforcement learning (DRL) has become more and more widespread. DRL has demonstrated great potential in addressing highly complex and challenging control and decision-making problems [14,15,16]. DRL can be divided into model-free approaches and model-based approaches. Since the states collected from the short-track speed skating simulation environment are discrete and fully observed, model-free approaches are well suited to decide which action to perform to maximize the reward when faced with a certain state.

With the success of DQN [17], more and more model-free approaches have been proposed, such as DDQN, Proximal policy optimization(PPO), A2C, A3C and other algorithms based on value function and policy gradient [18,19,20,21,22]. The mainstream application fields of DRL are robotics, neural machine translation, computer systems, etc. [23,24,25,26,27,28]. These industrial achievements have shown the effectiveness of the agent human-like decision-making. However, deep reinforcement learning has been less studied on the tactical decision-making problem of short track speed skating. Changpeng et al. [10] use a neural network to guide the agent to learn the correlation between state and action in the process of short-track speed skating. This method achieves more intelligent tactical decision-making ability than the traditional decision tree method, but over-reliance on prior knowledge leads to poor performance on the problem of going out of bounds.

Yang et al. [11] can improve the decision-making efficiency of agents through the DRL method to a certain extent. However, when the current state update is not available in that model, the lack of prior knowledge will cause the problem of the model to become complex, increasing the state space and slowing the convergence speed. In addition, the tactical decision as the action information in that article lacks a clear and scientific explanation of the setting.

The mainstream simulation environments used for deep reinforcement learning are generated by the gym toolkit or rendered by 3d engines such as unity3d and unreal engine [29,30,31]. To comprehensively save development costs and reduce computation, we use a scripted simulation environment to train agents.

## 3. Proposed Methods

### 3.1. Simulation Environment

The simulation environment used in this paper is mainly composed of three parts: the venue, the competition rules and the athletes. The venue simulation is to model the basic information of the venue used in the short track speed skating competition, including the length of the straight, the length of the curve, the radius of the inner and outer curves, the start position and the end position of 500 m short track speed skating competition. According to the standard field information of short track speed skating, we set up a simulated competition field in a two-dimensional rectangular coordinate system.

The simulation modeling of competition rules mainly includes the constraints that athletes should strictly observe. For example, when skating on a curve section with six markers, athletes should skate outside of the markers. Athletes must avoid collisions during the competition, and they should clearly meet the conditions for completing the race.

**Figure 1:**
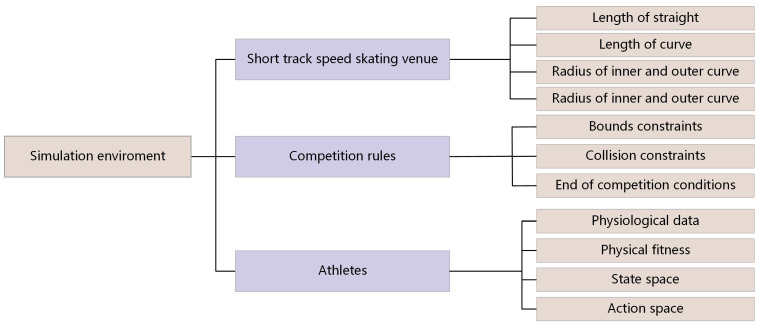
The construction framework of the simulation environment.

The simulation modeling of athletes is based on the basic physiological data of athletes, physiological fitness, state space and action space. The physiological data mainly include the height and weight of the athlete, the average windward area of the body while skating, and the upper and lower limits of the skating speed and acceleration. Physiological fitness is the embodiment of the residual endurance of athletes, and it is one of the key factors affecting the performance of athletes. The state space and action space contain the state data and action data of the athletes every 0.1 s. The overall simulation environment is shown in Figure 1:

### 3.2. State Space and Action Space

**Figure 2:**
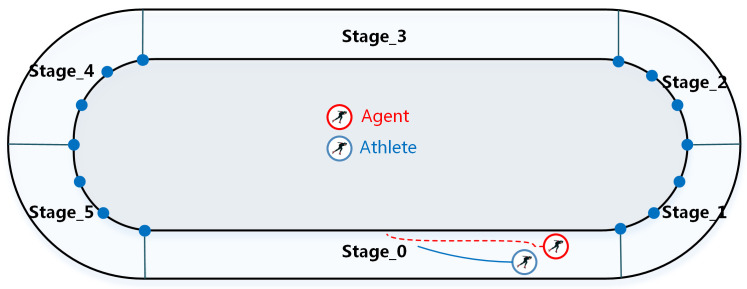
Schematic diagram of the six stages.

The state space mainly includes the two-dimensional coordinate information, ranking, physiological fitness, speed and the current stage. The action space contains motion direction *d* and acceleration *a*. After referencing a large amount of short track speed skating track data, we divide each lap of the race into six stages, as shown in Figure 2.

Reasonable restrictions on the exploration ability of the agent can accelerate the training speed of the model. Therefore, each time the agent selects an action, it is given a target direction to guide it to complete the current stage faster. The target points target and action space are defined as follows:

(1) $$target\in \left\{(7,-14.98),(15,-22.43),(23,-14.98),(23,14.98),(15,22.43),(7,14.98)\right\}$$

(2) $$a\in \left\{-2,-1,0,1,2,3,4,5\right\}$$

(3) $$d\in \left\{0,\frac{1}{24}\pi ,\frac{1}{12}\pi ,\frac{1}{6}\pi ,-\frac{1}{24}\pi ,-\frac{1}{12}\pi ,-\frac{1}{6}\pi \right\}$$

The state space at time *t* depends on the selection of the state space and action space of the agent at time t−1. The sampling interval *t* of trajectory information in this experiment is 0.1 s, then the update rule of the velocity $v_{t}$ at time *t* and direction *d* are as follows:

(4) $$v_{t}=v_{t-1}+a_{t-1}t$$

(5) $$d_{t}=d_{t-1}+d$$

The coordinate information at time t−1 is $\left(x_{t-1},y_{t-1}\right)$, and the direction and acceleration in the action space selected at this time are $d_{t-1}$ and $a_{t-1}$, respectively. Then the coordinate information $\left(x_{t},y_{t}\right)$ in the state space at time *t* is updated by the following rules:

(6) $$\left\{\begin{array}{c} x_{t}=x_{t-1}+\frac{1}{2}a_{t-1}\cos\left(d_{t-1}\right)t^{2}\\ y_{t}=y_{t-1}+\frac{1}{2}a_{t-1}\sin\left(d_{t-1}\right)t^{2} \end{array}\right.$$

Short track speed skaters need to correctly judge the physical energy consumption during the race. Konings [32] points out that the physical energy of short-track speed skaters to maintain speed or accelerate in the next few laps has an important impact on the final ranking of the race. Therefore, we design the physiological fitness to indirectly describe the remaining physical energy of the agent at different moments of the race. The speed of physiological fitness consumption mainly depends on the acceleration behavior of the agent and the amount of resistance that needs to be overcome during skating. The physical fitness ω is defined as follows:

(7) $$\omega =\omega_{ini}-△\omega$$

(8) $$△\omega =\left(\mu mg+\frac{1}{2}C\rho Sv^{2}\right)\times \sqrt{△x+△y}$$

$\omega_{ini}$ represents the initial physical fitness of each athlete. μ is the friction coefficient. *m* is the mass of the athlete. *g* is the acceleration of gravity. *C* is the coefficient of air resistance. ρ is the air density. *S* is the windward area of the athlete when skating. *v* is the instantaneous speed of the athlete, and △x and △y are the offsets of each dimension in the two-dimensional coordinate system in the sampling time, respectively.

### 3.3. Tactical Decision Model

**Figure 3:**
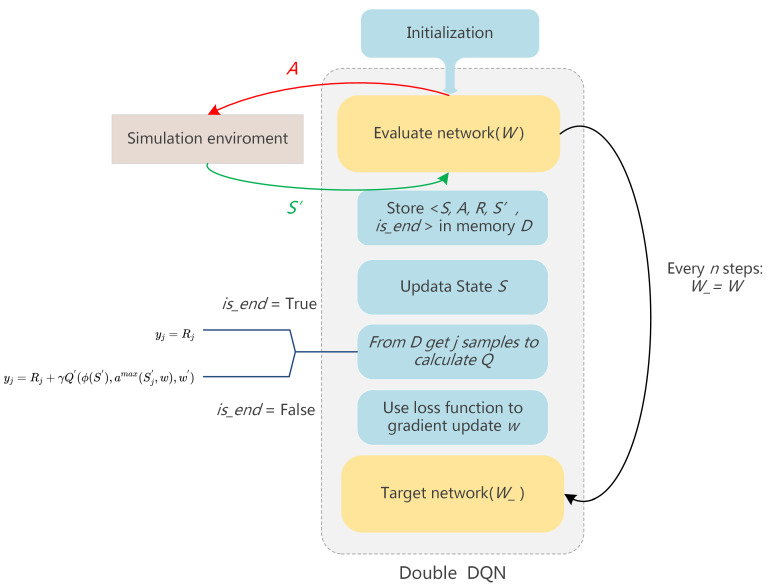
The structure of the Double DQN we used in this article.

In this paper, the agent uses the DDQN algorithm to learn tactical decisions for short-track speed skating. DDQN replaces the traditional Q-table with a neural network, so it has the ability to solve large-scale reinforcement learning problems. It simultaneously optimizes the two steps of decoupling the target Q-value action selection and the target Q-value calculation, so as to eliminate the problem of over-estimation. The structure of the proposed model is shown in the Figure 3.

The input of the model is the state vector ϕ(S) corresponding to our state *S*, and the output is the action-value function *Q* of all actions in that state. DDQN finds the action $A^{max}\left(S_{j}^{{}^{\prime }},W\right)$ corresponding to the maximum *Q* value in the evaluate network, and then uses this action to calculate the *Q* value which is the *y* for *j* samples.

(9) $$A^{max}\left(S_{j}^{{}^{\prime }},W\right)=\underset{A^{\prime }}{argmax}Q\left(\phi \left(S_{j}^{{}^{\prime }}\right),A,W\right)$$

(10) $$\left\{\begin{array}{cc} y_{j}=R_{j} & is\_end=True\\ y_{j}=R_{j}+\gamma Q^{{}^{\prime }}\left(\phi \left(S^{{}^{\prime }}\right),a^{max}\left(S_{j}^{{}^{\prime }},w\right),w^{{}^{\prime }}\right) & is\_end=False \end{array}\right.$$

$S_{j}$ represents the current state, and $S_{j}^{{}^{\prime }}$ represents the state after the agent performs action *A*. *W* and $W^{{}^{\prime }}$ are the parameters of the evaluate network and target network, respectively. γ is the decay factor. *R* is the reward obtained with the reward function. Then the model updates the network parameters *W* by optimizing the loss function Loss, and assigns the parameters of the evaluated network to the target network after updating the evaluation network every *n* time.

(11) $$Loss=\frac{1}{m}\sum_{j=1}^{m}{\left(y_{j}-Q\left(\phi \left(S_{j}\right),A_{j},W\right)\right)}^{2}$$

#### 3.3.1. Reward Function

The setting of the reward function directly affects how quickly the model maximizes the reward for action or training by Q-value [33]. Too many positive rewards make the agent tend to stop exploring more rewards, which weakens exploration that might lead to failure, and too many negative rewards cause training to terminate prematurely. In this paper, the main goal of the athlete is to complete the race in the shortest possible time while reaching the finish line and to try to reserve enough energy for the later laps to execute the tactic flexibly. Therefore, we design the stage reward $r_{stage}$, physiological fitness reward $r_{phy}$ and time reward $r_{time}$ to achieve the desired effect.

(12) $$r_{stage}=\left\{\begin{array}{cc} lap+r_{0} & stage_{next}=stage_{cur}+1\\ 0 & stage_{next}\neq stage_{cur}+1 \end{array}\right.$$

lap is the lap of the current race. $stage_{cur}$ and $stage_{next}$ denote the current stage and the next stage that the agent should be in, respectively.

(13) $$r_{phy}=sigmoid\left(lap\times \left(w_{ini}-△w\right)\right)$$

*w* is the physiological fitness defined in Equation (7), and we use sigmoid function to regulate the stabilization of this reward.

(14) $$r_{time}=\left\{\begin{array}{cc} -{\left(t-T_{ave}\right)}^{2} & t\geq T_{ave}\\ \;\;\;{\left(t-T_{ave}\right)}^{2} & t<T_{ave} \end{array}\right.$$

The time reward $r_{time}$ is mainly determined by the competition time *t* of the agent and the average competition time $T_{ave}$ of all athletes in that round. The total reward *r* is defined as follows:

(15) $$r=r_{stage}+r_{phy}+r_{time}$$

#### 3.3.2. Summary of Tactics

After full communication with the coach team, we summarize and analyze the behavior of the agent after training, and classify the short-track speed skating tactics of the agent and the athletes in the same experimental group based on each stage. The specific classification criteria are as follows:

Leading-skating tactic: the agent needs to do its best to become the first place as soon as possible at the current stage. If the agent is not currently in the first position, it needs to surpass to reach the leading position.

Following-skating tactic: if the agent is currently in the lead position, it continues to lead. If the agent is not in the first position, the opponent athlete in the previous position at the current instant should be the following target for the agent.

Full-speed tactic: the agent strives to reach the highest skating speed in the record in a short time. The agent is supposed to achieve the optimal situation of the current stage at the cost of a large amount of its physiological fitness.

Idle-speed tactic: the agent needs to control the speed to maintain good physiological fitness. This tactic is usually used to reserve the physiological fitness to execute other tactics when the situation appears to be deadlocked.

## 4. Results and Discussion

### 4.1. Experimental Composition and Settings

**Figure 4:**
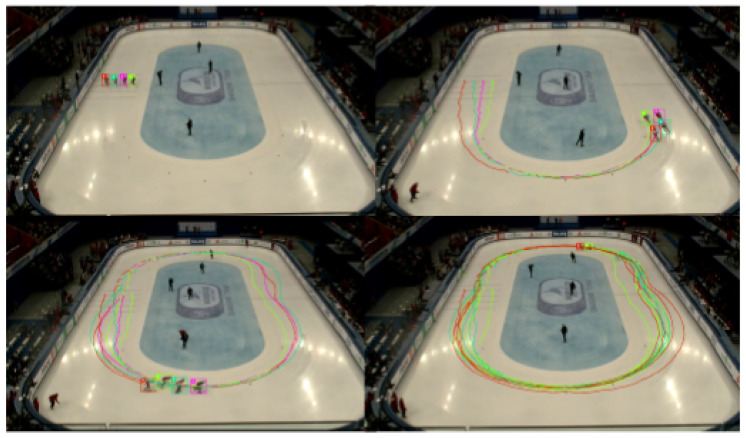
Visualized trajectories of teal short track speed skating competitions.

Based on the established simulation environment, we select 16 groups of real competition data of 500 m short track speed skaters as a data set and perform training for each competition. Specifically, in a short track speed skating competition, one athlete is randomly selected as the agent to replace him, and the remaining four athletes skate according to the real performance of the actual competition (Figure 4).

All experiments in this paper are based on NVIDIA GeForce TX 1060 5 GB graphics card, 256 G RAM and Intel(R) Core(TM) i9-10900X CPU @ 3.70 GHz. The simulation environment and tactical decision model are based on PyTorch 1.7.1. The parameters of our model are shown in Table 1.

### 4.2. Discussion

**Figure 5:**
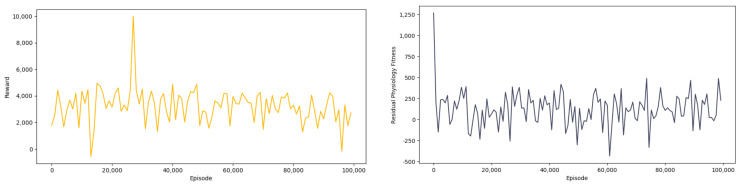
The reward chart and residual physiology fitness chart.

The reward chart and residual physiology fitness chart of the training are shown in Figure 5. It can be observed that through continuous learning and exploration, the reward obtained by the agent gradually converges from a poor reward value to a better reward value, and the agent can also allocate and use physical strength more scientifically in testing competition.

**Figure 6:**
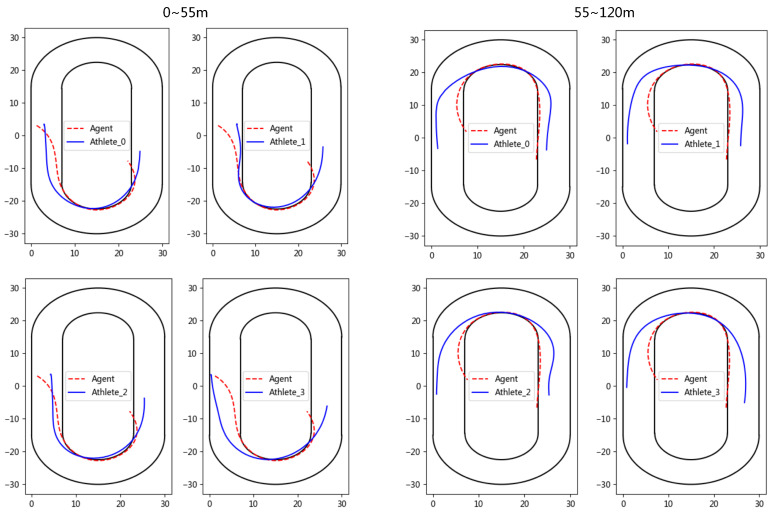
The skating trajectory of the agent and athletes in the simulation competition.

We randomly sample and visualize a group of test competition data for analysis, and mainly focus on the tactics of the first lap. As shown in Figure 6, it is clear that the agent is trained to enter the curve area and leave the curve area faster than the athlete, and the agent prefers to save energy in the straight and not surpass other athletes to gain the leading position. These data highlight the importance of the athlete effectively controlling the ankle and knee muscles during the curve section, as well as the ability of the athlete to anticipate body movements in advance.

**Figure 7:**
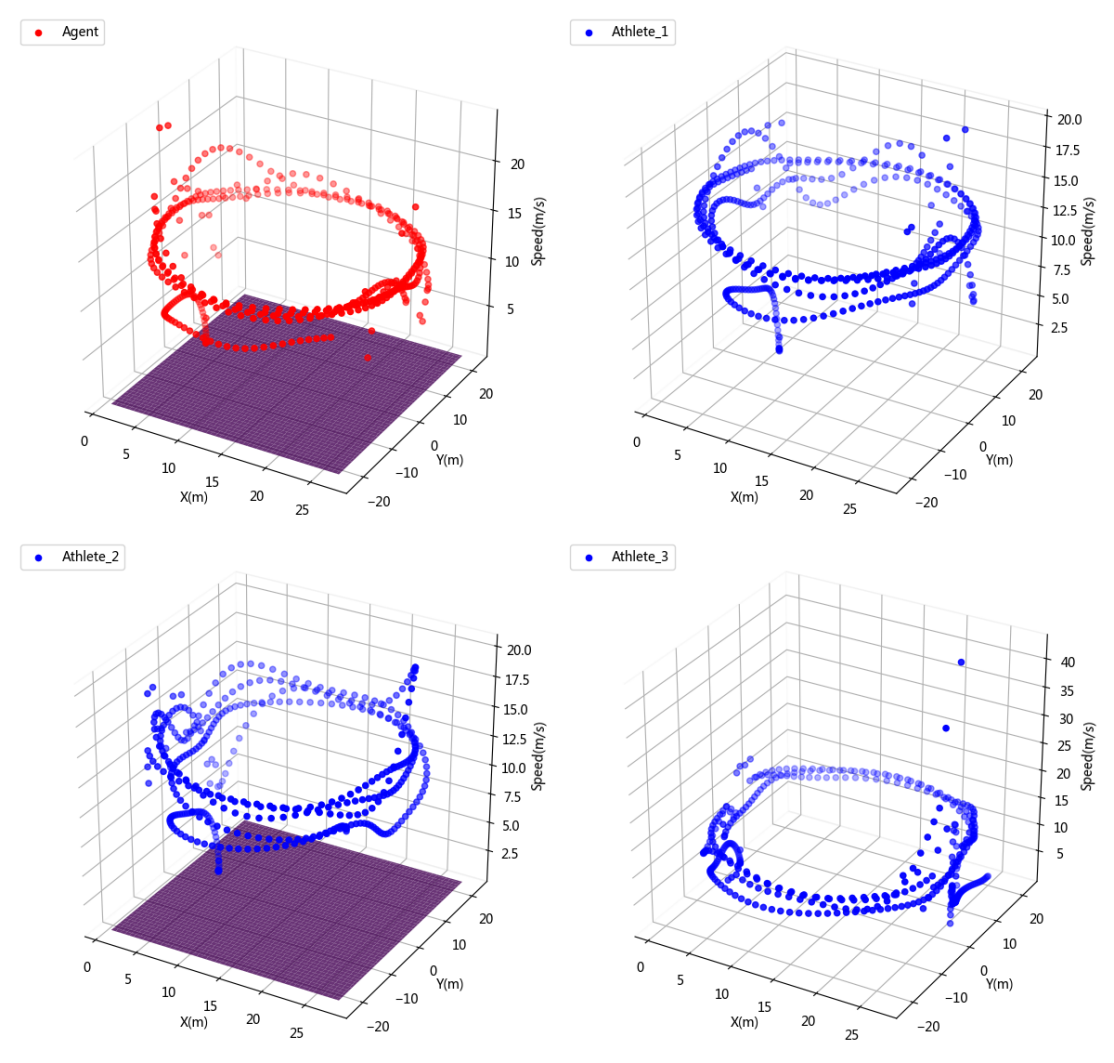
3D simulation of short track speed skating competition data.

In order to see the speed change of the athletes and agents in the competition more intuitively, we visualize the three-dimensional information of position information and speed as shown in Figure 7. It can be seen that both the agent and the athlete increase their speed to the fastest skating speed in the second lap, which proves that the learning of physical energy allocation by the agent is almost no different from professional athletes. On the other hand, this also proves the scientific nature of our reward setting and also verifies the rationality of the physiological fitness distribution of the agent.

In order to verify the effectiveness of our proposed tactical decision model, we use the data of the same athlete for testing and compare our method with the decision tree model, neural network model and previous tactical decision-making method [11]. The experimental results are shown in Table 2.

**Figure 8:**
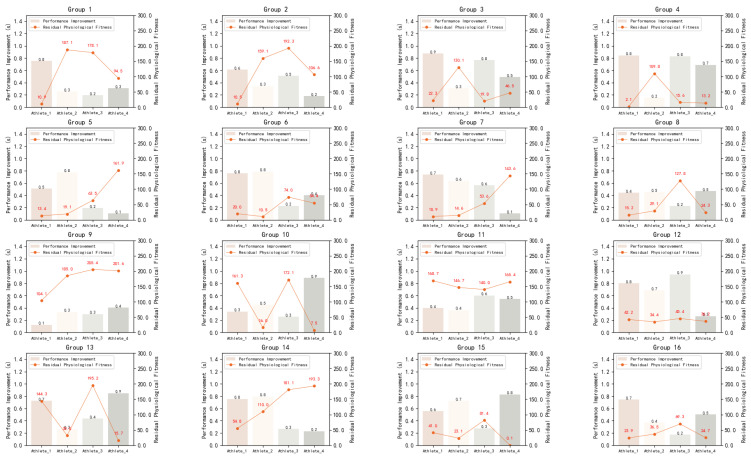
Comparison of the real crossing time between 16 groups of competition athletes and the corresponding agent.

In order to better reflect the efficiency of the tactical decision model on the performance improvement of athletes and the allocation of physiological fitness, we show the results of 16 groups of experiments in Figure 8, where the X-axis is the performance improvement of 4 athletes, and the Y-axis is the performance improvement in seconds and the residual physiological fitness. It can be clearly found that the performance-improved agent can use almost all the physiological fitness in the experiments compared with the athletes. However, the agent with less performance improvement could not use up all of its physiological fitness, which also shows that blindly saving physical strength without applying correct tactics will have an impact on the performance of the competition.

## 5. Conclusions

This paper mainly studies the tactical analysis and learning of short track speed skating based on deep reinforcement learning. We propose a tactical decision model for short track speed skating based on the DDQN model and guide the agent to learn better skating methods in the simulation environment. The trained skating behaviors are summarized into four skating strategies. 16 groups of experimental results show that our method can effectively improve the performance of athletes and physiological fitness. Compared with the decision tree, neural network and tactical decision-making method, our result is improved by 5.3 s, 3.7 s and 1.1 s, respectively. Our future work will focus on analyzing more data related to the muscle strength of athletes’ knee and ankle joints, as well as developing methods to assess athletes’ psychological resilience when facing major events. This will provide athletes with more intelligent and reliable guidance to improve their performance.

## Figures and Tables

**Table 1:** Experimental parameters setting.

Parameters	Value
Learning rate	0.0001
Episode	100,000
Batch size	100
Update frequency (*n*)	70
Memory capacity	100
Reward $r_{0}$	1
Initial physiological fitness ($W_{ini}$)	1700
Input/output dimension	5
Number of layers	8

**Table 2:** Results of comparative experiments.

Method	Best Record (s)	Average Record (s)
Decision tree	44.3	45.1
Neural network	42.7	43.8
Decision-making method [11]	40.1	43.0
Our method	39.0	40.2

## Data Availability

Due to the nature of this research, participants of this study did not agree for their data to be shared publicly, so supporting data are not available.
